# Redefining the high variable genes by optimized LOESS regression with positive ratio

**DOI:** 10.1186/s12859-025-06112-5

**Published:** 2025-04-15

**Authors:** Yue Xie, Zehua Jing, Hailin Pan, Xun Xu, Qi Fang

**Affiliations:** 1 College of Life Sciences, University of Chinese Academy of Sciences, Beijing, 100049 China; 2 BGI Research, Shenzhen, 518083 China; 3 BGI Research, Hangzhou, 310030 China

**Keywords:** Single cell transcriptome, High variable genes, Feature selection

## Abstract

**Background:**

Single-cell RNA sequencing allows for the exploration of transcriptomic features at the individual cell level, but the high dimensionality and sparsity of the data pose substantial challenges for downstream analysis. Feature selection, therefore, is a critical step to reduce dimensionality and enhance interpretability.

**Results:**

We developed a robust feature selection algorithm that leverages optimized locally estimated scatterplot smoothing regression (LOESS) to precisely capture the relationship between gene average expression level and positive ratio while minimizing overfitting. Our evaluations showed that our algorithm consistently outperforms eight leading feature selection methods across three benchmark criteria and helps improve downstream analysis, thus offering a significant improvement in gene subset selection.

**Conclusions:**

By preserving key biological information through feature selection, GLP provides informative features to enhance the accuracy and effectiveness of downstream analyses.

**Supplementary Information:**

The online version contains supplementary material available at 10.1186/s12859-025-06112-5.

## Background

Single-cell RNA sequencing (scRNA-seq) has profoundly transformed biological research, revealing cellular level transcriptomics profiles and heterogeneity [1–3]. In the routine analysis of RNA-seq data, the gene expression matrix needs to first go through a feature selection before most analytic steps (like dimension reduction, clustering or differential expression analysis) for computational efficiency, due to the ultra-high number of genes. A typical move is to select and keep a subset of genes whose expression has higher variance across all cells, known as the identification of highly variable genes (HVGs). Taking into account the variance of features was an intuitive solution in general data science, but more fundamentally, it is assumed that these variances tend to suggest its underlying biological significance, which usually ties to cellular heterogeneity, biological processes or molecular mechanisms [4].

Existing feature selection methods can generally be categorized into two types: those based on statistical or distributional models and those relying on clustering or graph-based approaches. The former includes methods such as VST [5], SCTransform [6], SCMarker [7], M3Drop and NBDrop [8]. The widely-used Seurat package offers VST [5] and SCTransform [6], which leverage a mean–variance relationship, instead of solely using variance. VST computes the variance through standardized expression level, whereas SCTransform utilizes pearson residuals derived from a generalized linear model to compute the variance. SCMarker [7] identifies genes with bimodal or multimodal expression distributions and those that exhibit co-expression or mutually exclusive expression patterns with other genes. M3Drop and NBDrop [8] are developed to leverage dropout-rates over variance. M3Drop fits a Michaelis–Menten function to model the relationship between mean expression levels and dropout rates and then employs a *t*-test to evaluate the significance of each gene, while NBDrop calculates the expected dropout rates of each gene through a negative binomial distribution and derives each gene’s *p*-value of observed dropout rates by a binomial model. In contrast, clustering or graph-based methods identify important genes by constructing gene–gene or cell–cell relationship networks and applying clustering or graph-theoretical techniques. FEAST [9] assesses gene significance using *F*-statistics derived from consensus clusters. HRG [10] constructs a cell–cell similarity network to select genes exhibiting regional expression patterns. Additionally, geneBasisR [11] selects genes with the maximum distance between the true manifold and the manifold constrcuted using the selected gene panel iteratively. Both CellBRF [12] and DELVE [13] rely on reconstructing the graph of cell neighborhoods using the k-nearest neighbors (k-NN) algorithm. The key distinction between these two approaches lies in their strategies for adjusting cell types which would be used to assess gene importance through expression variations across different cell types. CellBRF employs a combination of the synthetic minority over-sampling technique(SMOTE) [14] based oversampling and cluster center based undersampling to correct for cell type distribution imbalances, whereas DELVE employs kernel herding sketching [15] to effectively sample representative cell neighborhoods and eliminate redundant cells.

Despite the advancements in computational methods for gene feature selection, all existing approaches are fundamentally challenged by the high sparsity [16] and dropout noise that are characteristic of scRNA-seq data. This sparsity arises from a complex interplay between true biological expression selectivity and technical dropout artifacts, making it difficult to accurately model using simple statistical priors or predefined distributional assumptions. As a consequence, statistical and distributional model based methods often struggle to fully capture the heterogeneous nature of single-cell gene expression. While these models aim to account for biological and technical variability, their assumptions may not always align with the intricate data structure, leading to biases in feature selection. In particular, the prevalent dropout noise not only affects the identification of highly variable genes but also comprises the construction of gene–gene co-expression networks and cell–cell similarity graphs. This results in inaccurate correlation estimates, suboptimal clustering outcomes, and misleading representations in graph-based analyses. Given these challenges, there is a clear and pressing need for more robust feature selection strategies that can effectively distinguish biologically meaningful genes while mitigating the adverse effects of dropout noise and sparsity.

In this work, we introduce genes identified through LOESS with positive ratio (GLP), a novel feature selection method designed to identify biologically informative genes by examining the relationship between the positive ratio and average expression level. Instead of defining a dropout-rate, GLP utilizes the positive ratio of genes as a more straightforward yet precise estimator of the true population parameter [8]. Furthermore, the method integrates optimized LOESS regression [17], which leverages a local gene background as a reference, allowing it to capture nuanced non-linear relationships within specific subsets of data. A key strength of GLP is its use of the Bayesian Information Criterion (BIC) [18] to automatically determine the optimal bandwidth for the LOESS regression. This adaptive bandwidth selection ensures the model fits the data accurately, while avoiding overfitting, thereby enhancing the robustness of the feature selection process. Furthermore, GLP incorporates a two-step LOESS regression procedure. In the first step, it applies Tukey’s biweight robust statistical method [18, 19] to identify outlier genes. In the second step, these outliers are assigned zero weights to minimize their influence, ensuring more accurate detection of biologically relevant genes. We benchmarked GLP against eight state-of-the-art feature selection methods using 20 scRNA-seq datasets [20–35] (See Supplementary Table 1, Additional File 1) from diverse biological contexts, ranging from developmental studies to disease models. Surprisingly, Our comprehensive evaluation demonstrates that GLP consistently outperforms other methods across three benchmark criteria: adjusted rand index (ARI) [36], normalized mutual information (NMI) [37], and the silhouette coefficient [38]. In addition, we demonstrated the capability of GLP to enhance downstream analysis performance through specific data analyses, including differential gene expression analysis and trajectory inference. These findings highlight GLP’s potential to provide a more accurate and robust feature selection process for scRNA-seq data and thus can help improve downstream analysis. In summary, GLP represents a significant advancement in the precise selection of gene subsets, maximizing the preservation of biological information and thereby improving the outcomes of downstream analyses.

## Methods

### The core assumption of GLP

In recent years, several studies have investigated the biological implications of dropout rates and their relationship with expression levels [39–41]. Given that dropout rates provide a more accurate estimate of the true population parameter than variance [8], we were inspired by these findings to hypothesize that genes with higher expression levels at a given positive ratio are likely to be more significant and better reflect true biological information. To illustrate this hypothesis, we model scRNA-seq data using a random Poisson distribution with parameter $\lambda$ indicating gene average expression level for example. Given that the expression level of a gene is *X* and its positive ratio is *f*, we can derive the following inference:

1 $$f = P\left( {X \ge 1} \right) = 1 - e^{ - \lambda } ,$$

According to Taylor's theorem, Eq. (1) can be transformed into the following form:

2 $$\hat{f} \approx \lambda$$

Thus, when the parameter $\lambda$ is small, the average expression level of a gene can be approximated by its positive ratio. However, upon generalizing this relationship to all genes within real datasets, we observed that it transforms into a non-linear form, reflecting additional complexities introduced by technological or biological variability.

Given this non-linearity, we propose that it is more effective to identify the relationship between the positive ratio and the average expression levels locally rather than globally. Consequently, deviations where gene expression levels exceed these expected values are likely indicative of true biological differences rather than random variability. Therefore, we hypothesize that genes with expression levels significantly higher than expected are of greater importance and carry the most relevant biological information.

### Algorithm of GLP

**Fig. 1:**
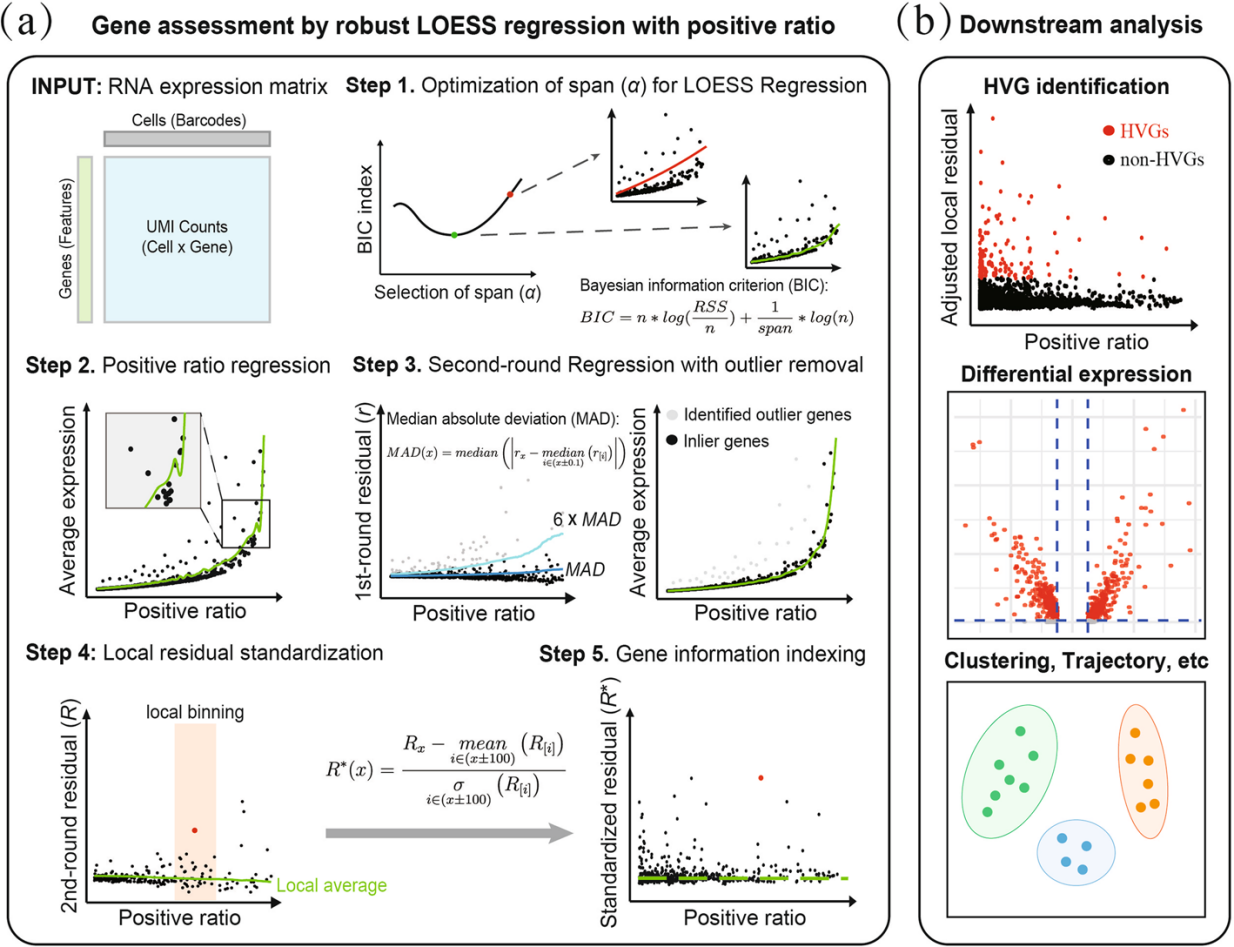
**a** Overview of the GLP workflow. GLP begins by taking the RNA expression matrix (genes × cells) as input. Step 1: The optimal span (*α*) for locally estimated scatterplot smoothing regression (LOESS) regression is selected based on the Bayesian Information Criterion(BIC). Step 2: A first-round LOESS regression is performed to model the relationship between the positive ratio and average expression. Step 3: Outliers are detected using Tukey’s biweight method, and a second-round LOESS regression is then conducted, excluding these outliers to improve robustness. Step 4: Residuals from the second-round regression are locally standardized. Step 5: Highly informative genes are identified based on each gene’s standardized residuals. **b** Downstream analysis. The gene sets derived from GLP can be applied to various downstream analyses, including identifying highly variable genes(HVGs), detecting differentially expressed genes, performing clustering, trajectory inference, etc.

The primary objective of GLP is to select a subset of genes with average expression levels larger than that predicted from positive ratios (Fig. 1a). GLP accepts expression counts matrix *g* × *c* as input, where *g* rows represent individual genes and *c* columns represent individual cells. Given a gene *j* with expression level *X* (a vector with length* c*) in *c* cells, we define average expression level $\lambda$ and positive ratio* f* for gene *j* as:

3 $$\lambda_{j} = \frac{1}{c}\sum\limits_{i = 1}^{c} {X_{ij} } ,$$

4 $$f_{j} = \frac{1}{c}\sum\limits_{i = 1}^{c} {\min \left( {1,\,X_{ij} } \right)}$$

Subsequently, we constructed a two-dimensional distribution of all genes within the input matrix, where *f* as the independent variable and $\lambda$ as the dependent variable. It is noteworthy that genes captured in less than 3 cells were filtered out and excluded from further analysis.

Prior to executing the LOESS regression, we utilized the BIC to adaptively determine the optimal smoothing parameter for the LOESS regression model (*⍺*):

5 $$RSS = \sum\limits_{j = 1}^{g} {\left( {y_{j} - \hat{y}_{j} } \right)}$$

6 $$BIC = c \times In\left( \frac{RSS}{c} \right) + k \times In\left( c \right)$$

where *c* represents the number of observations, and *k* is the degrees of freedom in Eq. (6).

Noticeably, through testing on real single-cell data, we observed that the two-dimensional distribution of genes based on the positivity ratio and mean expression level exhibits an approximately linear relationship in regions with low positivity ratios, while the curvature increases as the positivity ratio grows. This suggests that in regions with higher positivity ratios, the expression patterns of genes become more complex, necessitating the selection of appropriate *⍺* values to achieve more accurate curve fitting. At the same time, considering that the gene count in most mammals typically ranges from 20,000 to 30,000, we empirically defined the range of *⍺* from 0.01 to 0.1 with a step size of 0.01. Specifically, an *⍺* of 0.1 corresponds to a local regression window incorporating approximately 2,000 genes, while values exceeding this threshold may fail to adequately capture the underlying data structure. Conversely, an *⍺* of 0.01 corresponds to a local window of approximately 200 genes, with smaller values potentially leading to overfitting (see Supplementary Fig. 1a–d, Additional File 2). Therefore, the selected range and step size of *⍺* are carefully designed to achieve an optimal balance between capturing fine-scale variations and mitigating overfitting risks across diverse datasets, while simultaneously reducing computational complexity, and enhancing processing efficiency. To objectively determine the most appropriate *⍺*, we evaluate the BIC for each preset value, selecting the *⍺* that minimizes BIC as the optimal regression parameter. Additionally, we also support user-defined *⍺* values to better accommodate specific needs.

With the optimal *⍺* established, we proceed to employ a two-round LOESS regression model to fit the relationship between positivity ratio and average expression levels. This model excels at capturing local characteristics of the data, making it particularly well-suited for the exploratory analysis of complex nonlinear relationships.

The first round of regression utilizes Tukey’s biweight robust statistical method to identify outlier genes. According to our criteria, outliers are defined as genes exhibiting residuals exceeding six times the local median absolute deviation (MAD)[42] within a range of 0.1 on either side. In the second round of regression, we assign a weight of zero to the identified outliers and to genes with positive ratios greater than 0.99. This approach effectively mitigates their influence on the model, thereby enhancing the robustness and reliability of the analysis.

By establishing a well-fitted two-round LOESS regression model, we can predict the expected average expression levels at varying positivity ratios and obtain the residuals for each gene relative to these expected values. The basic form of the LOESS regression model is:

7 $$\hat{\lambda }_{j} = \sum\limits_{k = 1}^{n} {\theta_{k} \left( {f_{i} ,\alpha } \right) \times \lambda_{k} }$$

where $\lambda_{j}$ represents the expected average expression level for gene *j* with positive ratio being *f*_*j*_, *θ*_*k*_*(f*_*j*_*, ⍺)* is the weight calculated based on the distance and span parameter *⍺* for gene *j*. The span parameter *⍺* determines the proportion of genes used in each local regression, controlling the trade-off between bias and variance. After that, we calculate the residuals for each gene, which are the differences between the observed and expected average expression levels:

8 $$e_{j} = \lambda_{j} - \hat{\lambda }_{j} ,$$

where *e*_*j*_ is the residual for the* j*-th gene, $\lambda_{j}$ is the observed expression level.

Since the range of residuals is related to the positivity ratio and increases with higher positivity ratios, we standardize the residuals from Eq. (8) for each gene using a window of 100 genes on either side of the gene to calculate the Z-scores of the residuals:

9 $$Z_{j} = \frac{{e_{j} - \mu }}{\sigma }$$

Here, *Z*_*j*_ is the Z-score of the residual for the *j*-th gene, *μ* and *σ* is the mean and standard deviation of the residuals of genes within the window. This standardization ensures the comparability of residuals across all genes.

Ultimately, GLP ranks the genes in descending order based on the magnitude of their standardized residuals, selecting and outputting the top 1,000 genes (by default) with the highest residual Z-scores. These selected genes can subsequently be utilized for various downstream analyses, including the identification of HVGs, differential expression analysis, clustering, and differentiation trajectory analysis (Fig. 1b).

### scRNA-seq datasets

We collected 20 publicly available single-cell datasets [20–35] (See Supplementary Table 1, Additional File 1) from various sources, including the NCBI Gene Expression Omnibus (GEO), Cell x Gene Data Portal, and the Single Cell Portal. These datasets span diverse biological systems, such as the immune system, bone marrow, mouse brain, and tumors. To reduce noise and enhance the robustness of our algorithm evaluation, we excluded cell types represented by only a single cell. Importantly, each dataset contains not only raw sequencing matrices but also annotated cell type labels, facilitating comprehensive evaluation and comparison across different methods.

### Data preprocess

scRNA-seq data were all processed and analyzed using the Seurat package[43] (version 4.3.0.1) in R (4.1.0). The raw count matrix was first used to create a Seurat object, and normalization was performed to account for differences in sequencing depth. Specifically, the data were normalized using the NormalizeData function, followed by scaling to correct for gene-specific variation using the ScaleData function, considering all genes available in the dataset.

### Dimensionality reduction and clustering

For dimensionality reduction, principal component analysis (PCA) was performed with a subset of HVGs calculated by each algorithm to capture the maximum variance in the data. The Shared Nearest Neighbor (SNN) graph was constructed using the 20 nearest neighbors and 10 principal components as input. Clustering was performed using the Louvain algorithm [44], which was selected due to its computational efficiency in handling large-scale networks and its ability to automatically determine the optimal number of communities through modularity optimization, particularly suitable for identifying distinct cell populations in single-cell datasets. The resolution parameter was adjusted to achieve an identical number of clusters corresponding to the known cell types in each dataset. Uniform Manifold Approximation and Projection (UMAP) was generated using the RunUMAP function.

### Differential gene expression analysis

Differentially expressed genes between the identified clusters were determined using Seurat's FindAllMarkers function, with default parameters (min.pct = 0.1, logfc.threshold = 0.25), based on normalized expression values and assessed using the Wilcoxon rank sum test.

### Trajectory Inference

Trajectory analysis was performed using the slingshot package [45] (version 1.8.0), which enables the inference of cellular trajectories based on selected HVGs and existing UMAP. Default parameter settings were used to infer the pseudotemporal ordering and differentiation trajectories.

### Dimensional reduction and clustering performance evaluation criteria

Here, we employed external evaluation metrics, including ARI [36] and NMI [37], to quantify the concordance between the unsupervised clustering outcomes and the known cell types. Additionally, we utilized the Silhouette Coefficient [38] as an internal evaluation metric to assess the compactness and separation of the clusters in the feature space.

These metrics provide a comprehensive assessment of the clustering performance, thereby enabling a robust comparison of the efficacy of different gene selection algorithms.

## Results

### GLP outperforms eight state-of-art methods across twenty datasets

We compared GLP with eight state-of-the-art feature selection algorithms, including VST [5], SCTransform [6], SCMarker [7], M3Drop, NBDrop [8], FEAST [9], HRG [10], and genebasisR [11]. For VST, SCTransform, FEAST and genebasisR, we set the number of selected genes to 2000, 3000, 2000, and 50, respectively according to their default settings. The number of genes selected by the other methods was determined by their respective algorithms. All other parameters for these methods were also maintained at their default settings.

To evaluate the gene sets selected by these methods, we applied dimensionality reduction and unsupervised clustering to each gene set on 20 collected datasets (See Supplementary Table 1, Additional File 1). We hypothesize that gene sets preserving a higher degree of biological signal and minimizing noise will lead to superior clustering performance, closely resembling true cell types.

**Fig. 2:**
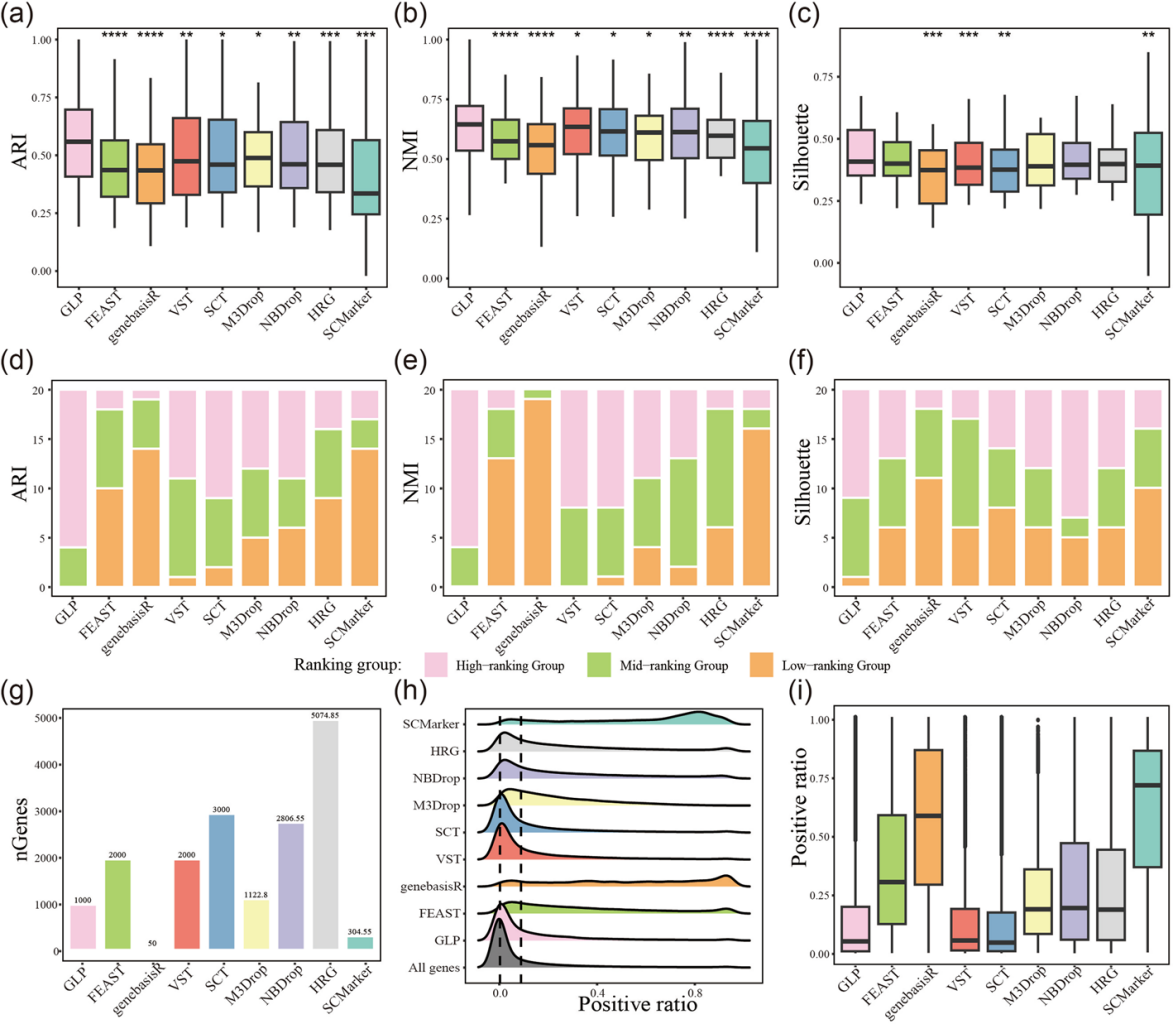
Benchmark GLP against existing feature selection methods across 20 datasets. (**a**–**c**) Performance comparison of all methods in single-cell clustering in terms of adjusted rand index(ARI), normalized mutual information (NMI) and the silhouette coefficient. (**d**–**f**) Distribution of method rankings across all datasets. Methods are categorized into high-ranking (positions 1–3), mid-ranking (positions 4–6), and low-ranking (positions 7–9) groups. (**g**) The average gene set size derived from each method. (**h**) Positive ratio distribution of all genes and gene sets selected by each method, with black dashed lines indicating thresholds at 0.01 and 0.1. (**i**) Boxplot showing the positive ratio of gene sets derived by each method, highlighting the variation in gene selection across methods

We evaluated the performance of these nine gene selection algorithms on 20 collected datasets. Their effectiveness in dimensionality reduction and clustering was assessed using ARI, NMI, and silhouette coefficient metrics. It is important to emphasize that, to ensure comparability of clustering results across various feature selection methods, the number of clusters was aligned with the known cell annotation types in each dataset by adjusting the resolution parameter during the clustering process (See Supplementary Table 2, Additional File 1). Through the Wilcoxon test, we show that GLP demonstrated significantly superior performance compared to other methods in terms of both ARI and NMI metrics (Fig. 2a–b) (See Supplementary Table 3–4, Additional File 1). The median ARI and NMI scores for GLP were 0.54 and 0.62, respectively, with a median improvement of 15.5% and 3% over the second high-scoring methods. In terms of silhouette coefficient, the GLP method demonstrated a significant advantage over the other four methods, with the exception of FEAST, M3Drop, NBDrop and HRG (Fig. 2c) (See Supplementary Table 5, Additional File 1). We categorized the ranking performance across datasets into three groups: high-ranking (positions 1–3), mid-ranking (positions 4–6), and low-ranking (positions 7–9). Remarkably, in terms of ARI and NMI metrics, GLP demonstrated outstanding performance, with over 80% of datasets classified in the high-ranking group, while the remaining datasets fell into the mid-ranking group, with none in the low-ranking group. Similarly, for the Silhouette metric, GLP showed robust results, with over 80% of datasets positioned in either the high-ranking or mid-ranking groups, and only a small fraction in the low-ranking group(Fig. 2d–f). More specifically, GLP demonstrates superior performance, ranking among the top three algorithms in terms of ARI, NMI, and silhouette coefficient on 16, 17, and 11 out of 20 datasets.

An intriguing observation is that the GLP gene set comprises only 1000 genes to achieve optimal performance (Fig. 2g) (See Supplementary Table 6, Additional File 1). This indicates that GLP achieves superior clustering performance with a more streamlined gene set. Furthermore, not only does GLP exhibit superior robustness and efficiency, it also closely mirrors the distribution characteristics of positivity ratios observed in single-cell data, particularly capturing a greater number of low positivity-ratio genes as well as VST and SCT (Fig. 2h–i). This alignment offers the significant advantage that GLP identifies HVGs without being biased by the positive ratio or expression level of the genes, making them more representative of the entire genome. Moreover, with its robust process, GLP mitigates the effects of technical noise, which is especially pertinent in single-cell datasets, and enhances the robustness of downstream analyses, such as clustering, dimensionality reduction, and functional enrichment, leading to more reliable and biologically meaningful insights.

In summary, the comprehensive evaluation of these metrics indicates that GLP outperforms other algorithms in identifying biologically relevant genes.

### GLP facilitates improved downstream analysis in human telencephalic organoid scRNA-seq data

To scrutinize the biological effectiveness of genes selected by GLP, we selected a dataset of human telencephalic organoids [29] (See Supplementary Table 1, Additional File 1) to provide a detailed demonstration of how GLP enhances downstream analyses..

**Fig. 3:**
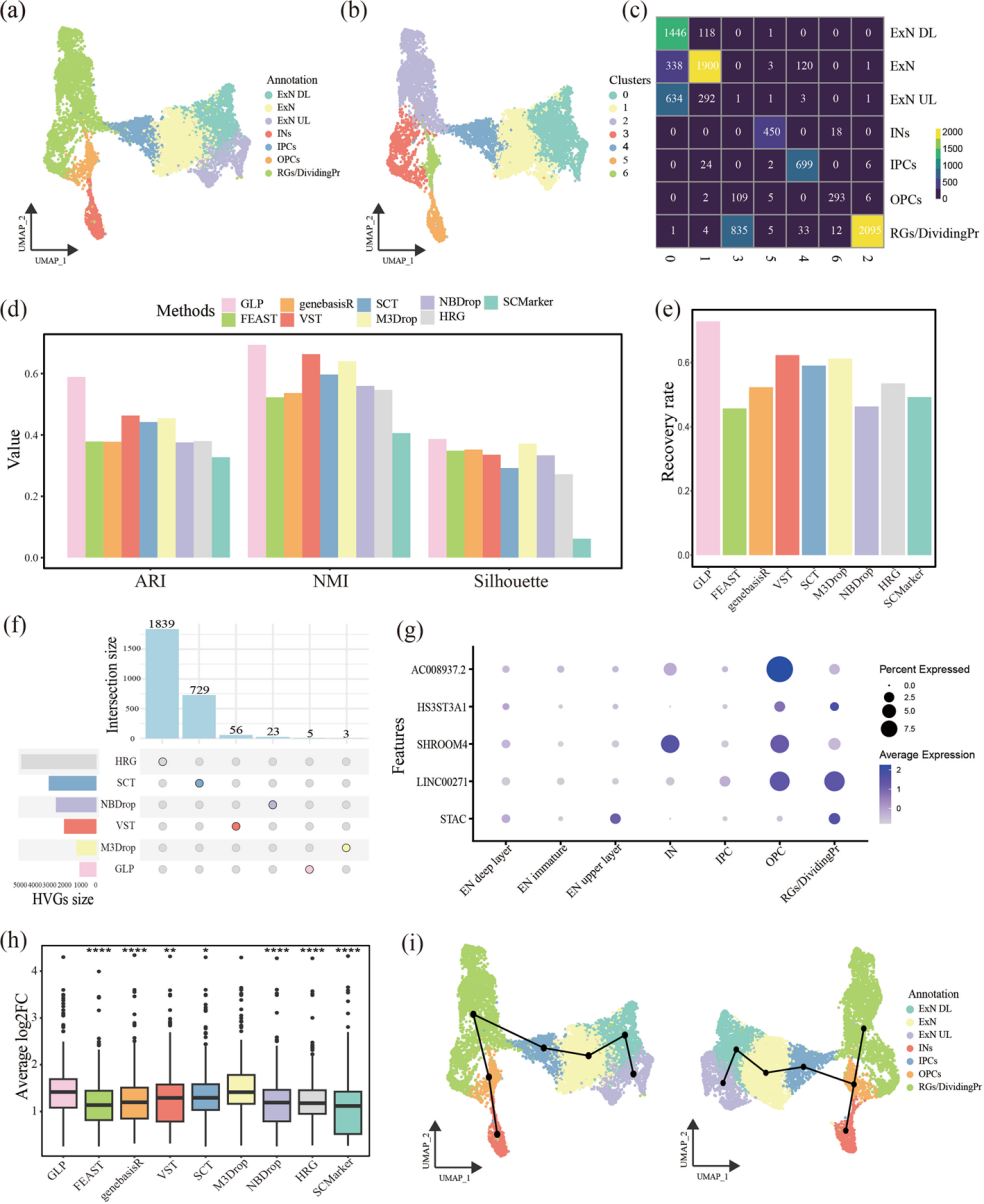
Case study of GLP. (**a**–**b**) UMAP visualization of human telencephalic organoid scRNA-seq data using features selected by GLP. (**a**) UMAP projection colored by annotated cell types, including radial glia dividing progenitors (RGs/DividingPr), oligodendrocyte precursor cells (OPCs), interneurons (INs), intermediate progenitor cells (IPCs), immature excitatory neurons (ExN), deep-layer excitatory neurons (ExN DL) and upper-layer excitatory neurons (ExN UL). (**b**) UMAP projection colored by unsupervised clustering results. (**c**) Confusion matrix comparing clustering results using GLP-selected features. (**d**) Comparison of clustering performance across methods based on three evaluation criteria. (**e**) Number of correctly matched cells in clustering using features selected by each method. (**f**) Size of the unique gene set identified by each method. (**g**) Dotplot showing expression levels of five genes uniquely selected by GLP. (**h**) Box plot of the average log2 fold-change (log2FC) of the top 50 differential expression genes, computed based on clustering results from each method. (**i**) Trajectory inference based on HVGs, with UMAP projections derived from GLP (left) and VST (right), respectively

First, GLP selected genes can help improve the results of dimensionality reduction and clustering. On this dataset, GLP outperforms other algorithms in terms of ARI, NMI, and silhouette coefficient, with respective values of 0.585, 0.689, and 0.384 (Fig. 3d). To provide a more intuitive comparison of how accurately cells were classified into known categories, we examined the UMAP spatial distribution of the unsupervised clustering results of each algorithm against the known cell types (Fig. 3a–b) (See Supplementary Fig. 2a–h, Additional File 2). We also calculated the corresponding confusion matrices based on the relationship between the unsupervised clustering results and the known cell annotations (Fig. 3c) (See Supplementary Fig. 3a–h, Additional File 2). By adjusting the rows and columns of the confusion matrices according to the correct category correspondences, we defined the recovery rate as the sum of the diagonal elements divided by the total number of cells. A higher recovery rate indicates a more accurate reconstruction of the known cell types. Among the methods, GLP achieved the highest recovery rate at 72.8%, followed by VST and M3Drop at 62.3% and 61.2%, respectively (Fig. 3e).

It is noteworthy that all methods classified radial glia dividing progenitors (RGs/DividingPr) into more than one category, with GLP distinguishing it into two categories. Upon review of the original data, we found that this cell group indeed comprises two cell types: radial glial progenitor cells and actively dividing neural progenitor cells. Based on classical marker genes, GLP’s unsupervised clustering accurately corresponded to these two cell types (See Supplementary Fig. 3i, Additional File 2). Specifically, Tenascin C (TNC) and HOP Homeobox (HOPX) are highly expressed in radial glial cells with stemness properties [46]; hes family bHLH transcription factor 1 (HES1) is highly expressed in neural stem cells and plays a crucial role in inhibiting differentiation and maintaining stem cell state [47]; marker of proliferation Ki-67 (MKI67) is a widely recognized gene associated with high cellular proliferation [48]. Additionally, under the constraint of a limited number of clusters, no method was able to distinguish deep-layer excitatory neurons (ExN DL) and upper-layer excitatory neurons (ExN UL) in a single iteration. However, when the resolution was increased, these two types could be successfully identified (See Supplementary Fig. 3j–k, Additional File 2). Thus, GLP demonstrates superior capability in aiding downstream clustering analysis and enhancing clustering performance compared to other methods.

More interestingly, as previously mentioned, GLP is capable of generating a more streamlined yet information-rich gene set. In this particular dataset, GLP utilized only 1000 genes, including 5 genes uniquely identified by GLP (Fig. 3f), which exhibited significant differential expression across the known annotated groups (Fig. 3g). This indicates that the genes selected by GLP possess a higher degree of biological information and lower noise signal compared to those selected by other methods.

Second, GLP aids in the identification of genes with more distinct differential expression characteristics. To demonstrate this, we calculated the differentially expressed genes (DEGs) for each cluster based on the unsupervised clustering results mentioned earlier, and extracted the top 50 genes. The box plot (Fig. 3h) shows that the median of the average log2 fold-change (log2FC) for the top 50 DEGs identified by GLP is higher than that of all other methods, and significantly higher than all methods except M3Drop. This suggests that the highly variable genes identified by GLP contribute to more accurate cell classification, thus facilitating the identification of genes with substantial differential expression in downstream analyses. Consequently, GLP’s selection of high-variance genes enhances the sensitivity of differential gene expression analysis, making it a valuable tool for uncovering biologically relevant genes in complex datasets.

Third, GLP was shown to significantly improve the performance of downstream trajectory inference analyses. To validate its advantage, we compared GLP with the widely used VST algorithm, focusing on their performance in trajectory inference. Specifically, we take HVGs identified by each method and along with UMAP dimensionality reduction results as inputs for trajectory inference using the Slingshot algorithm [45].

In particular, GLP accurately identified that intermediate progenitor cells (IPCs) are derived from RGs/DividingPr rather than oligodendrocyte precursor cells (OPCs) [49] (Fig. 3i). This precise reconstruction offers deeper insights into the dynamic process of cell differentiation and further validates GLP's potential in enhancing the accuracy of trajectory inference.

The results revealed that the HVGs identified by GLP better reflected changes in cellular states and differentiation paths. With GLP's support, the trajectory inference successfully reconstructed the differentiation relationships between cells, particularly improving the identification of branching points and developmental trajectories within cell subpopulations. In contrast, while VST performed well in general data processing, it did not provide sufficient assistance in trajectory inference, resulting in poorer reconstruction of cell differentiation trajectories.

In summary, the GLP algorithm significantly improves downstream analyses, particularly in dimensionality reduction, DEGs detection and trajectory inference tasks, through its optimized feature selection method and more accurate identification of HVGs. This advantage offers a powerful tool for researchers aiming to extract maximal biological insight from complex datasets.

### GLP balances longer runtime with enhanced accuracy

**Fig. 4:**
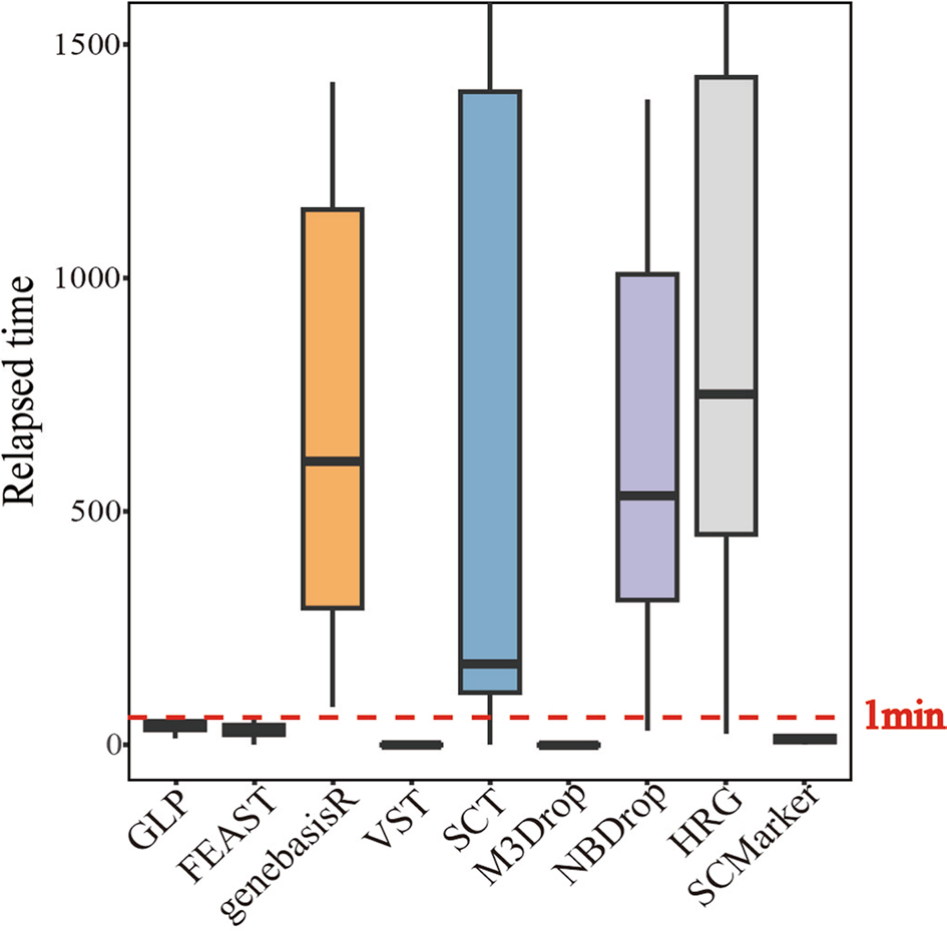
Comparison of runtime performance across different feature selection methods. The red dashed line represents the 1-min threshold

To evaluate the computational efficiency of GLP, we conducted a comprehensive analysis of the performance of all tested algorithms across 20 test datasets (Fig. 4). Among these algorithms, M3Drop emerged as the fastest, with average runtimes not exceeding 3 s. In contrast, GLP exhibited an average runtime of 46.97 s, ranking fifth in terms of speed. Notably, within the scope of all datasets, GLP exceeded an operation time of 1 min for only 4 out of the 20 datasets, with the longest duration recorded at 104 s. The primary factor contributing to GLP’s relatively longer runtime is the computation of the optimal value for the LOESS span width, a crucial step that enhances the accuracy of the results.

In summary, although GLP demonstrates a longer runtime when compared to algorithms such as FEAST, VST, M3Drop, and SCMarker, its execution time remains efficient, with most datasets requiring no more than 1 min for processing. This runtime is deemed acceptable for single-cell data analysis, considering the complexity and richness of the data involved. Therefore, GLP stands out as a practical and efficient tool for researchers, balancing computational efficiency with the need for robust analytical capabilities.

## Discussion

In this work, we developed GLP, a novel feature selection method specifically designed for scRNA-seq data. We have demonstrated that GLP is a robust and efficient approach for identifying HVGs from tens of thousands of candidates that minimizes noise and maximizes informational content, thereby enhancing the effectiveness of downstream analyses such as dimensionality reduction, clustering, DEGs detection and trajectory inference. The method’s strengths lie in its ability to capture both globally relevant features and subtle local variations, while effectively minimizing the influence of outliers.

As demonstrated in our results, existing feature selection methods, including VST and SCTransform, fail to accurately identify HVGs, resulting in inferior performance in dimensionality reduction, clustering, differential gene expression analysis, and trajectory inference when compared to GLP. This is primarily due to their reliance on statistical models and their limited ability to address dropout noise effectively. Among all the methods, M3Drop [11] performs at a moderately high level. Interestingly, M3Drop and GLP are conceptually similar, as both model the relationship between the positive ratio and the average expression level of genes. However, their implementations differ significantly: M3Drop employs the Michaelis–Menten equation to fit the relationship between these variables, whereas GLP leverages an optimized LOESS regression to adaptively and robustly capture the relationship between them. This difference makes the M3Drop model relatively less flexible, particularly when dealing with complex single-cell data. More specifically, our results show that M3Drop identifies fewer HVGs in the low positive ratio range, which results in an increased rate of false negatives. In contrast, GLP's adaptive approach effectively addresses low positive ratio scenarios, ensuring that the detection rate of HVGs is unaffected by positive ratio biases and resulting in a more representative set of HVGs.

GLP overcomes these challenges by integrating a novel approach that combines the positivity ratio and LOESS regression. By using the positivity ratio to capture the proportion of non-zero expression values for each gene, GLP provides a more robust measure of gene activity. Furthermore, GLP’s two-step LOESS regression model adds an extra layer of refinement, allowing for more accurate predictions of expected gene expression levels. Additionally, Tukey’s biweight method incorporated in the second round of regression ensures that outliers do not disproportionately affect the results, thereby enhancing the method’s robustness.

Another key innovation of GLP is its use of the Bayesian Information Criterion (BIC) to automatically determine the optimal span for LOESS regression. It can eliminate the need for manual parameter tuning, making the method more user-friendly and adaptable to a variety of datasets. The BIC-based approach ensures that the method remains flexible, while still maintaining optimal performance across diverse single-cell datasets.

Despite its strengths, GLP is not without limitations. One notable challenge is its computational complexity. LOESS regression, while powerful and flexible, can be computationally expensive, particularly when applied to large datasets with many genes and cells. To address this, future versions of GLP could benefit from computational optimizations, such as parallel processing or approximations of the LOESS model, to improve efficiency and scalability for large-scale studies. Moreover, the current version of GLP selects a fixed number of HVGs, which may not always align with the needs of specific studies. Users are encouraged to adjust the number of HVGs based on the magnitude of the standardized residuals, tailoring the gene selection process to suit their specific dataset.

In conclusion, GLP represents a significant advancement in the field of feature selection for scRNA-seq data. By integrating the positivity ratio, LOESS regression, and robust outlier handling, GLP provides a powerful tool for identifying HVGs and thus helps improve the accuracy of downstream analyses such as dimensionality reduction, clustering, DEGs detection and trajectory inference while minimizing the impact of dropout noise and sparsity. GLP’s ability to balance robustness and sensitivity makes it a valuable addition to the repertoire of single-cell analysis tools.

## Supplementary Information

Additional file 1.

Additional file 2.

## Data Availability

This paper analyzes existing, publicly available data, the detailed information please see supplementary Table 1, additional file 1.

## Abbreviations

ARI: Adjusted rand index
BIC: Bayesian information criterion
DEGs: Differentially expressed genes
ExN: Immature excitatory neurons
ExN DL: Deep-layer excitatory neurons
ExN UL: Upper-layer excitatory neurons
GEO: Gene expression omnibus
GLP: Genes identified through LOESS with positive ratio
HES1: Hes family bHLH transcription factor 1
HOPX: HOP Homeobox
HVGs: Highly variable genes
INs: Interneurons
IPCs: Intermediate progenitor cells
k-NN: K-nearest neighbors
LOESS: Locally estimated scatterplot smoothing
Log2FC: Log2 fold-change
MAD: Median absolute deviation
MKI67: Marker of proliferation Ki-67
NMI: Normalized mutual information
OPCs: Oligodendrocyte precursor cells
PCA: Principal component analysis
RGs/DividingPr: Radial glia dividing progenitors
scRNA-seq: Single-cell RNA sequencing
SMOTE: Synthetic minority over-sampling technique
SNN: Shared Nearest Neighbor
TNC: Tenascin C
UMAP: Uniform manifold approximation and projection
