# Supplementary material for: Redefining the high variable genes by optimized LOESS regression with positive ratio
Source: BMC Bioinformatics. 2025 Apr 15;26:104. doi: 10.1186/s12859-025-06112-5 (PMC12001687; doi:10.1186/s12859-025-06112-5)
Supplement: Supplementary file 1 — Additional file 1. [file 12859_2025_6112_MOESM1_ESM.docx]

| Name | Repositories | Accession Number | nCells | nGenes | nTypes | Description | Reference |
| --- | --- | --- | --- | --- | --- | --- | --- |
| Arcelus | GEO | GSE124731 | 2036 | 33694 | 7 | Innate T cells | [12] |
| Engel | GEO | GSE74596 | 203 | 23341 | 4 | NKT cells | [13] |
| Eroglu | GEO | GSE180914 | 2386 | 18140 | 15 | Salamander heart | [14] |
| Heng | GEO | GSM3580745 | 3931 | 27998 | 12 | Mouse brain | [15] |
| Kornberg | GEO | GSM8006467 | 4514 | 36601 | 3 | Duodenum | [16] |
| Lambo1 | GEO | GSM7494257 | 6755 | 24343 | 19 | Bone marrow | [17] |
| Lambo2 | GEO | GSM7494258 | 4476 | 24343 | 18 |  |  |
| Lambo3 | GEO | GSM7494259 | 5151 | 24343 | 17 |  |  |
| Ma1 | GEO | GSM5709304 | 747 | 25885 | 7 | Liver cancer | [18] |
| Ma2 | GEO | GSM5709306 | 923 | 25885 | 7 |  |  |
| Mangelinck | GEO | GSE242428 | 38624 | 20869 | 6 | CD4+ cells | [19] |
| Manno | GEO | GSE76381 | 1715 | 18539 | 17 | Mouse brain | [20] |
| Martins | GEO | GSE220085 | 9458 | 19592 | 7 | Telencephalic organoids | [21] |
| Ohinata | GEO | GSE159183 | 736 | 36040 | 4 | Stem cell lines | [22] |
| Piau1 | GEO | GSM7011521 | 2845 | 32738 | 28 | Embroid Body | [23] |
| Piau2 | GEO | GSM7011525 | 3931 | 32738 | 28 |  |  |
| Rosen | GEO | GSM5820698 | 4701 | 25180 | 14 | White adipose | [24] |
| Savas | Broad Single Cell Portal | SCP2331 | 5759 | 31246 | 10 | Breast cancer | [25] |
| [Siletti](https://doi.org/10.1126/science.add7046) | Cellxgene | 470565f2-5afc-456a-b617-18e4496c04fd.cxg | 4717 | 59357 | 11 | Human brain | [26] |
| Szabo | Cellxgene | eeacb0c1-2217-4cf6-b8ce-1f0fedf1b569.cxg | 9337 | 33244 | 8 | PBMC | [27] |

**Supplementary Table 1.** Detailed information of the datasets used in this study.

**Supplementary Table 2.** Clustering resolution for each method across all datasets.

| Data | FEAST | genebasisR | HRG | HVG | GLP | M3Drop | NBDrop | SCMarker | SCT |
| --- | --- | --- | --- | --- | --- | --- | --- | --- | --- |
| Arcelus | 0.83 | 1.3 | 0.8 | 1 | 1.1 | 0.55 | 0.85 | 0.2 | 1 |
| Engel | 0.3 | 0.9 | 0.2 | 0.3 | 0.3 | 0.055 | 0.3 | 0.6 | 0.2 |
| Eroglu | 1.2 | 1 | 0.9 | 0.85 | 0.9 | 1 | 0.9 | 1 | 1.1 |
| Heng | 0.48 | 0.8 | 0.45 | 0.18 | 0.4 | 0.5 | 0.25 | 0.5 | 0.4 |
| Kornberg | 0.1 | 0.1 | 0.08 | 0.06 | 0.06 | 0.1 | 0.08 | 0.03 | 0.06 |
| Lambo1 | 1.3 | 1.4 | 1.4 | 1.2 | 1.3 | 1.32 | 1.3 | 1.7 | 1.4 |
| Lambo2 | 1.31 | 1.75 | 1.4 | 1.3 | 1.5 | 1.2 | 1.5 | 1.7 | 1 |
| Lambo3 | 1 | 1.5 | 1.1 | 0.9 | 1 | 1.2 | 1.1 | 1.5 | 1 |
| Ma1 | 0.5 | 0.3 | 0.3 | 0.6 | 0.35 | 0.2 | 0.6 | 0.62 | 0.4 |
| Ma2 | 0.3 | 0.4 | 0.3 | 0.36 | 0.4 | 0.16 | 0.15 | 0.3 | 0.13 |
| Mangelinck | 0.1 | 0.17 | 0.16 | 0.15 | 0.13 | 0.15 | 0.15 | 0.2 | 0.15 |
| Manno | 1.83 | 2.5 | 1.45 | 1.5 | 1.7 | 1.8 | 1.335 | 1.7 | 1.64 |
| Martins | 0.25 | 0.4 | 0.25 | 0.21 | 0.21 | 0.228 | 0.25 | 0.3 | 0.2 |
| Ohinata | 0.08 | 0.4 | 0.3 | 0.2 | 0.1 | 0.2 | 0.2 | 0.2 | 0.3 |
| Piau1 | 3.1 | 3.8 | 3.3 | 3.4 | 3.9 | 3.9 | 3.4 | 3.6 | 3.2 |
| Piau2 | 3 | 3.4 | 2.9 | 2.1 | 2.4 | 2.3 | 2.3 | 3.1 | 2.3 |
| Emont | 0.54 | 0.8 | 0.4 | 0.53 | 0.5 | 0.5 | 0.45 | 0.85 | 0.5 |
| Savas | 0.5 | 0.793 | 0.35 | 0.4 | 0.4 | 0.5 | 0.35 | 0.275 | 0.33 |
| Siletti | 0.2 | 0.3 | 0.18 | 0.2 | 0.3 | 0.14 | 0.3 | 0.25 | 0.2 |
| Szabo | 0.15 | 0.2 | 0.1 | 0.1 | 0.06 | 0.13 | 0.15 | 0.1 | 0.1 |

**Supplementary Table 3.** Comparison of GLP and Eight State-of-the-Art Gene Selection Methods for Single-Cell Clustering Based on Adjusted Rand Index (ARI).

| Data | FEAST | genebasisR | HRG | HVG | GLP | M3Drop | NBDrop | SCMarker | SCT |
| --- | --- | --- | --- | --- | --- | --- | --- | --- | --- |
| Arcelus | 0.502 | 0.362 | 0.462 | 0.446 | 0.491 | 0.359 | 0.429 | 0.081 | 0.435 |
| Engel | 0.417 | 0.19 | 0.456 | 0.241 | 0.487 | 0.169 | 0.22 | 0.237 | 0.238 |
| Eroglu | 0.556 | 0.566 | 0.524 | 0.729 | 0.743 | 0.599 | 0.599 | 0.643 | 0.653 |
| Heng | 0.812 | 0.834 | 0.804 | 0.939 | 0.925 | 0.814 | 0.928 | 0.77 | 0.923 |
| Kornberg | 0.186 | 0.108 | 0.177 | 0.189 | 0.192 | 0.476 | 0.189 | -0.021 | 0.188 |
| Lambo1 | 0.294 | 0.317 | 0.312 | 0.335 | 0.32 | 0.319 | 0.315 | 0.345 | 0.342 |
| Lambo2 | 0.234 | 0.219 | 0.236 | 0.245 | 0.247 | 0.256 | 0.242 | 0.248 | 0.248 |
| Lambo3 | 0.243 | 0.169 | 0.269 | 0.312 | 0.31 | 0.242 | 0.262 | 0.217 | 0.28 |
| Ma1 | 0.535 | 0.512 | 0.529 | 0.6 | 0.716 | 0.537 | 0.644 | 0.545 | 0.737 |
| Ma2 | 0.455 | 0.493 | 0.596 | 0.613 | 0.602 | 0.501 | 0.643 | 0.462 | 0.48 |
| Mangelinck | 0.26 | 0.208 | 0.345 | 0.291 | 0.428 | 0.437 | 0.416 | 0.261 | 0.334 |
| Manno | 0.354 | 0.411 | 0.401 | 0.382 | 0.419 | 0.41 | 0.396 | 0.279 | 0.426 |
| Martins | 0.376 | 0.375 | 0.377 | 0.46 | 0.585 | 0.451 | 0.373 | 0.325 | 0.439 |
| Ohinata | 0.993 | 0.986 | 0.993 | 1 | 1 | 1 | 0.993 | 1 | 1 |
| Piau1 | 0.33 | 0.318 | 0.327 | 0.345 | 0.374 | 0.368 | 0.39 | 0.31 | 0.376 |
| Piau2 | 0.396 | 0.458 | 0.448 | 0.488 | 0.537 | 0.551 | 0.524 | 0.396 | 0.511 |
| Emont | 0.639 | 0.608 | 0.668 | 0.668 | 0.691 | 0.723 | 0.686 | 0.626 | 0.611 |
| Savas | 0.507 | 0.48 | 0.548 | 0.571 | 0.58 | 0.601 | 0.493 | 0.114 | 0.598 |
| Siletti | 0.589 | 0.595 | 0.647 | 0.658 | 0.636 | 0.522 | 0.513 | 0.544 | 0.655 |
| Szabo | 0.915 | 0.541 | 0.932 | 0.898 | 0.912 | 0.616 | 0.932 | 0.76 | 0.93 |

**Supplementary Table 4.** Comparison of GLP and Eight State-of-the-Art Gene Selection Methods for Single-Cell Clustering Based on Normalized Mutual Information (NMI).

| Data | FEAST | genebasisR | HRG | HVG | GLP | M3Drop | NBDrop | SCMarker | SCT |
| --- | --- | --- | --- | --- | --- | --- | --- | --- | --- |
| Arcelus | 0.544 | 0.443 | 0.502 | 0.533 | 0.541 | 0.441 | 0.523 | 0.113 | 0.523 |
| Engel | 0.487 | 0.343 | 0.518 | 0.422 | 0.547 | 0.29 | 0.382 | 0.353 | 0.403 |
| Eroglu | 0.677 | 0.706 | 0.673 | 0.807 | 0.796 | 0.745 | 0.738 | 0.74 | 0.759 |
| Heng | 0.853 | 0.843 | 0.861 | 0.933 | 0.918 | 0.857 | 0.918 | 0.823 | 0.916 |
| Kornberg | 0.253 | 0.135 | 0.236 | 0.263 | 0.267 | 0.496 | 0.253 | 0.013 | 0.26 |
| Lambo1 | 0.506 | 0.501 | 0.508 | 0.526 | 0.522 | 0.519 | 0.514 | 0.533 | 0.532 |
| Lambo2 | 0.458 | 0.429 | 0.454 | 0.464 | 0.461 | 0.475 | 0.458 | 0.432 | 0.478 |
| Lambo3 | 0.46 | 0.401 | 0.478 | 0.509 | 0.51 | 0.474 | 0.476 | 0.43 | 0.493 |
| Ma1 | 0.661 | 0.646 | 0.653 | 0.708 | 0.718 | 0.675 | 0.703 | 0.65 | 0.749 |
| Ma2 | 0.57 | 0.57 | 0.662 | 0.661 | 0.658 | 0.594 | 0.674 | 0.577 | 0.586 |
| Mangelinck | 0.399 | 0.335 | 0.43 | 0.413 | 0.476 | 0.497 | 0.47 | 0.396 | 0.424 |
| Manno | 0.594 | 0.586 | 0.615 | 0.612 | 0.633 | 0.627 | 0.617 | 0.507 | 0.618 |
| Martins | 0.519 | 0.533 | 0.543 | 0.659 | 0.689 | 0.636 | 0.556 | 0.403 | 0.593 |
| Ohinata | 0.989 | 0.978 | 0.989 | 1 | 1 | 1 | 0.989 | 1 | 1 |
| Piau1 | 0.58 | 0.548 | 0.581 | 0.594 | 0.613 | 0.612 | 0.619 | 0.559 | 0.616 |
| Piau2 | 0.604 | 0.635 | 0.644 | 0.677 | 0.683 | 0.701 | 0.69 | 0.628 | 0.687 |
| Emont | 0.718 | 0.681 | 0.711 | 0.726 | 0.736 | 0.769 | 0.734 | 0.69 | 0.696 |
| Savas | 0.517 | 0.462 | 0.567 | 0.571 | 0.583 | 0.601 | 0.533 | 0.174 | 0.617 |
| Siletti | 0.642 | 0.648 | 0.66 | 0.693 | 0.674 | 0.611 | 0.61 | 0.646 | 0.688 |
| Szabo | 0.81 | 0.6 | 0.841 | 0.769 | 0.873 | 0.66 | 0.843 | 0.731 | 0.844 |

**Supplementary Table 5.** Comparison of GLP and Eight State-of-the-Art Gene Selection Methods for Single-Cell Clustering Based on Silhouette Coefficient.

| Data | FEAST | genebasisR | HRG | HVG | GLP | M3Drop | NBDrop | SCMarker | SCT |
| --- | --- | --- | --- | --- | --- | --- | --- | --- | --- |
| Arcelus | 0.481 | 0.356 | 0.44 | 0.328 | 0.368 | 0.218 | 0.38 | 0.205 | 0.305 |
| Engel | 0.607 | 0.428 | 0.639 | 0.489 | 0.585 | 0.325 | 0.453 | 0.497 | 0.498 |
| Eroglu | 0.426 | 0.45 | 0.396 | 0.454 | 0.482 | 0.496 | 0.478 | 0.514 | 0.449 |
| Heng | 0.538 | 0.559 | 0.606 | 0.66 | 0.672 | 0.585 | 0.673 | 0.555 | 0.677 |
| Kornberg | 0.386 | 0.241 | 0.401 | 0.386 | 0.383 | 0.455 | 0.391 | -0.052 | 0.375 |
| Lambo1 | 0.405 | 0.338 | 0.342 | 0.365 | 0.411 | 0.336 | 0.393 | 0.383 | 0.353 |
| Lambo2 | 0.285 | 0.235 | 0.282 | 0.309 | 0.305 | 0.28 | 0.285 | 0.213 | 0.28 |
| Lambo3 | 0.26 | 0.142 | 0.251 | 0.234 | 0.238 | 0.258 | 0.277 | 0.15 | 0.264 |
| Ma1 | 0.448 | 0.441 | 0.342 | 0.317 | 0.377 | 0.549 | 0.463 | 0.417 | 0.479 |
| Ma2 | 0.526 | 0.467 | 0.619 | 0.53 | 0.555 | 0.522 | 0.571 | 0.61 | 0.439 |
| Mangelinck | 0.395 | 0.392 | 0.362 | 0.382 | 0.416 | 0.372 | 0.398 | 0.401 | 0.389 |
| Manno | 0.353 | 0.231 | 0.412 | 0.243 | 0.29 | 0.258 | 0.399 | 0.306 | 0.22 |
| Martins | 0.346 | 0.35 | 0.27 | 0.333 | 0.384 | 0.369 | 0.331 | 0.061 | 0.29 |
| Ohinata | 0.876 | 0.856 | 0.9 | 0.817 | 0.887 | 0.848 | 0.833 | 0.849 | 0.876 |
| Piau1 | 0.248 | 0.155 | 0.267 | 0.241 | 0.251 | 0.245 | 0.275 | 0.201 | 0.274 |
| Piau2 | 0.221 | 0.229 | 0.28 | 0.27 | 0.253 | 0.323 | 0.313 | 0.176 | 0.263 |
| Emont | 0.452 | 0.457 | 0.479 | 0.482 | 0.529 | 0.518 | 0.5 | 0.434 | 0.447 |
| Savas | 0.369 | 0.242 | 0.369 | 0.415 | 0.405 | 0.407 | 0.358 | 0.112 | 0.377 |
| Siletti | 0.503 | 0.535 | 0.45 | 0.598 | 0.613 | 0.531 | 0.514 | 0.57 | 0.625 |
| Szabo | 0.378 | 0.453 | 0.407 | 0.421 | 0.426 | 0.482 | 0.342 | 0.561 | 0.322 |

**Supplementary Table 6.** The number of genes selected by each methods across all datasets.

| Data | FEAST | genebasisR | HRG | HVG | GLP | M3Drop | NBDrop | SCMarker | SCT |
| --- | --- | --- | --- | --- | --- | --- | --- | --- | --- |
| Arcelus | 2000 | 50 | 1746 | 2000 | 1000 | 40 | 73 | 6 | 3000 |
| Engel | 2000 | 50 | 915 | 2000 | 1000 | 14 | 4475 | 761 | 3000 |
| Eroglu | 2000 | 50 | 4443 | 2000 | 1000 | 2448 | 10485 | 815 | 3000 |
| Heng | 2000 | 50 | 5714 | 2000 | 1000 | 754 | 1775 | 285 | 3000 |
| Kornberg | 2000 | 50 | 1901 | 2000 | 1000 | 405 | 496 | 36 | 3000 |
| Lambo1 | 2000 | 50 | 4968 | 2000 | 1000 | 1387 | 1508 | 112 | 3000 |
| Lambo2 | 2000 | 50 | 4968 | 2000 | 1000 | 947 | 1029 | 74 | 3000 |
| Lambo3 | 2000 | 50 | 3975 | 2000 | 1000 | 1162 | 1168 | 97 | 3000 |
| Ma1 | 2000 | 50 | 1057 | 2000 | 1000 | 369 | 1138 | 205 | 3000 |
| Ma2 | 2000 | 50 | 1057 | 2000 | 1000 | 380 | 876 | 143 | 3000 |
| Mangelinck | 2000 | 50 | 13629 | 2000 | 1000 | 2186 | 4924 | 451 | 3000 |
| Manno | 2000 | 50 | 2643 | 2000 | 1000 | 785 | 1250 | 56 | 3000 |
| Martins | 2000 | 50 | 4798 | 2000 | 1000 | 1203 | 2536 | 37 | 3000 |
| Ohinata | 2000 | 50 | 25743 | 2000 | 1000 | 2561 | 12993 | 1850 | 3000 |
| Piau1 | 2000 | 50 | 1337 | 2000 | 1000 | 972 | 1814 | 176 | 3000 |
| Piau2 | 2000 | 50 | 3779 | 2000 | 1000 | 1263 | 1924 | 205 | 3000 |
| Emont | 2000 | 50 | 5807 | 2000 | 1000 | 1705 | 2703 | 237 | 3000 |
| Savas | 2000 | 50 | 2551 | 2000 | 1000 | 323 | 617 | 18 | 3000 |
| Siletti | 2000 | 50 | 5716 | 2000 | 1000 | 2074 | 2349 | 317 | 3000 |
| Szabo | 2000 | 50 | 4750 | 2000 | 1000 | 1478 | 1998 | 210 | 3000 |
