# Supplementary material for: Redefining the high variable genes by optimized LOESS regression with positive ratio
Source: BMC Bioinformatics. 2025 Apr 15;26:104. doi: 10.1186/s12859-025-06112-5 (PMC12001687; doi:10.1186/s12859-025-06112-5)
Supplement: Supplementary file 2 — Additional file 2. [file 12859_2025_6112_MOESM2_ESM.docx]

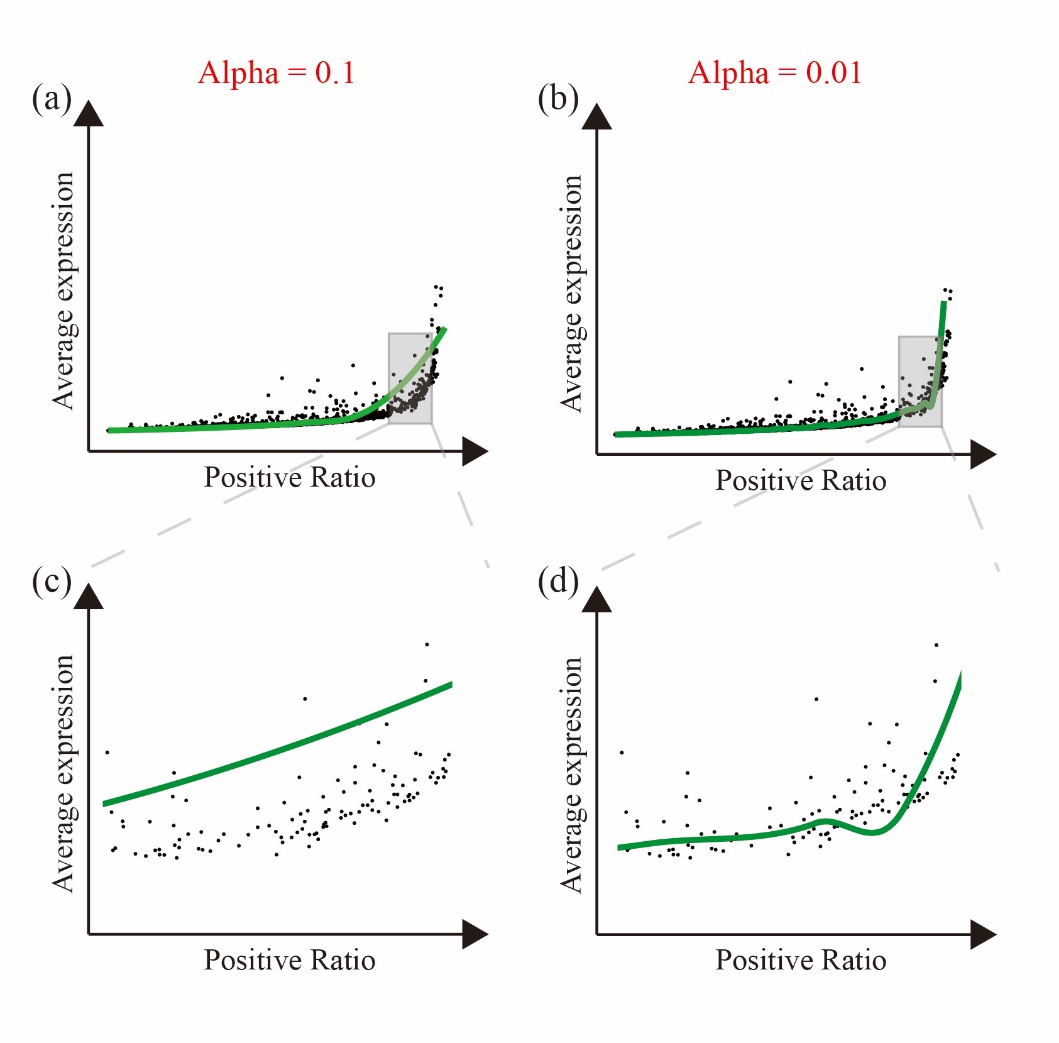


Supplementary Figure1. (a-b) Scatter plots depicting the relationship between average expression and positive ratio, fitted using LOESS regression with different *α* values. (a) *α* = 0.1; (b) *α* = 0.01. The green curve represents the fitted trend, while the gray shaded region marks a specific area for detailed comparison. (c, d) Magnified views of the shaded regions in (a) and (b), respectively, illustrating the local fitting differences.


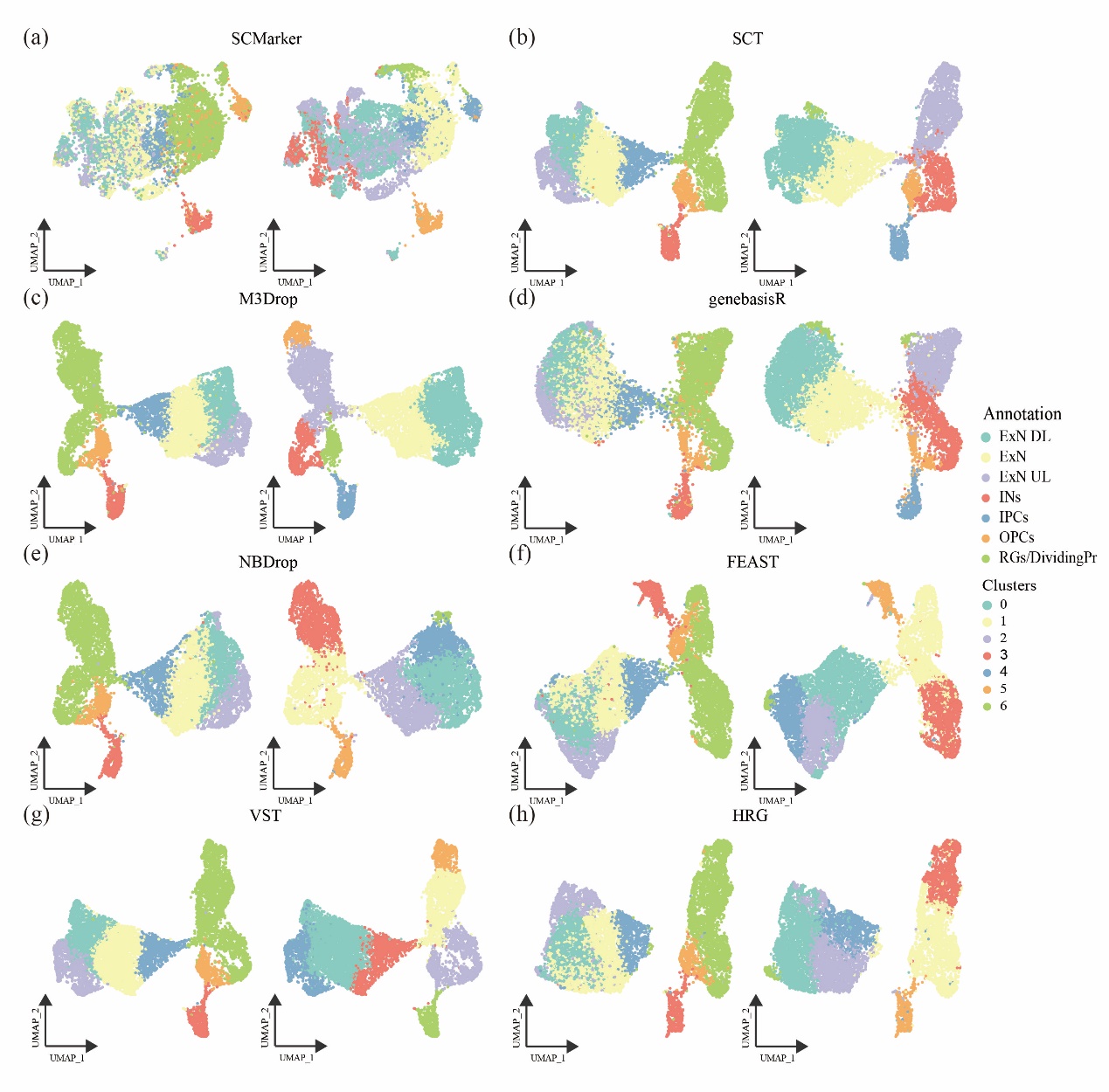


Supplementary Figure2. (a-h) UMAP visualization of human telencephalic organoid scRNA-seq data using features selected by each method. The left panels show annotated cell types, while the right panels display unsupervised clustering results for each method.


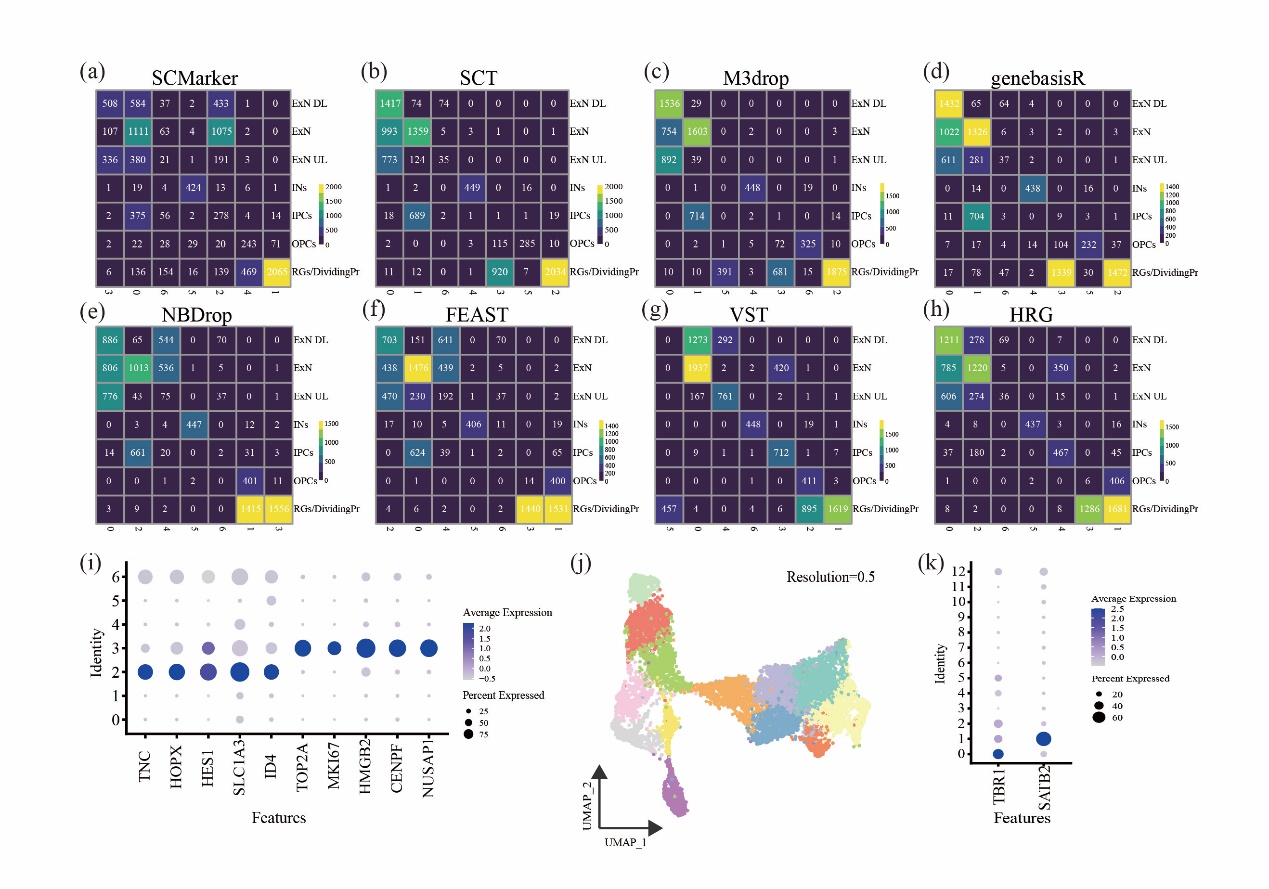


Supplementary Figure 3. (a-h) Confusion matrices comparing clustering results using features selected by each method. (i) Dotplot showing the expression of genes indicating radial glial progenitor cells and actively dividing neural progenitor cells. (j) UMAP visualization of human telencephalic organoid scRNA-seq data using features selected by GLP, with clustering resolution set to 0.5. (k) Dotplot showing the expression of genes indicative of deep-layer excitatory neurons (ExN DL) and upper-layer excitatory neurons (ExN_UL).
